# Effectiveness of an Internet-Based Acceptance and Commitment Therapy Intervention for Reducing Psychological Distress in Health Care Professionals: Randomized Controlled Trial

**DOI:** 10.2196/59093

**Published:** 2024-12-18

**Authors:** Lin Zhang, Shuang Huang, Sha Liu, Yuanxiu Huang, Shan Chen, Jinsong Hu, Mingzhong Xu

**Affiliations:** 1 Changsha Municipal Center for Disease Control and Prevention Changsha China

**Keywords:** acceptance and commitment therapy, internet-based intervention, stress, anxiety, depression, burnout, health care professionals, randomized controlled trial

## Abstract

**Background:**

Psychological distress is prevalent among health care professionals and can lead to poor-quality patient care. Internet-based acceptance and commitment therapy (iACT) is a promising intervention for improving mental health due to its low cost and easy access. However, there is limited evidence of its effectiveness in reducing health care professionals’ psychological distress.

**Objective:**

This study aims to examine the effects of iACT on psychological distress (stress, anxiety, and depression) among health care professionals in China.

**Methods:**

From October 2022 to February 2023, a total of 108 health care professionals were recruited via WeChat and randomized into a 6-week iACT intervention program with therapist support (n=54) or waitlist control group (n=54). The intervention included 21 self-guided sessions combining teaching videos, mindfulness practices, and journal writing, followed by 7 live conferences to share experiences and discuss questions, all conducted via WeChat. Primary outcomes (stress, anxiety, and depression) and secondary outcomes (burnout and psychological flexibility) were collected using the Sojump platform, the most popular web-based survey platform in China. Generalized estimating equations were used to compare the outcomes between groups and assess the effects of group, time, and group-by-time interaction. Subgroup and sensitive analyses were performed to test the robustness of our findings across various groups.

**Results:**

Among the 108 health care professionals, 68 (63%) completed the follow-up assessment at week 10, including 35 (64.8%) in the iACT group and 33 (61.1%) in the waitlist control group. Of the 54 participants in the iACT group, all attended at least 2 sessions, and 25 attended all 28 sessions. On average, participants attended 20 (71%) sessions. The iACT group showed significant improvement in the Depression Anxiety and Stress Scales-21 total score (*d*=0.82, 95% CI 0.39-1.26), and the effects were sustained for 4 weeks after the intervention (*d*=1.08, 95% CI 0.57-1.59). Compared to the control group, the iACT group showed significantly lower scores in burnout at week 6 (*d*=1.42, 95% CI 0.95-1.89) and week 10 (*d*=1.52, 95% CI 0.98-2.06). The iACT group showed significantly higher psychological flexibility at week 6 (*d*=1.23, 95% CI 0.77-1.69) and week 10 (*d*=1.15, 95% CI 0.64-1.66).

**Conclusions:**

The iACT effectively decreased health care professionals’ psychological distress and burnout and improved their psychological flexibility. Our findings provide implications and guidance for the development and broad implementation of iACT in health care settings to improve the mental health of health care professionals.

**Trial Registration:**

Chinese Clinical Trial Register ChiCTR2400093584; https://tinyurl.com/38werwsk

## Introduction

Worldwide, health care professionals experience significant psychological distress due to the demanding health care environment, heavy clinical workload, zero tolerance for mistakes, and conflicting roles, coupled with the shortage of resources in low-income countries [1]. Psychological distress refers to a state of emotional distress due to exposure to a stressful event that threatens one’s physical or mental health and the inability to cope effectively with this stressor, which eventually leads to a range of emotional turmoil, such as stress, depression, and anxiety [2]. The COVID-19 pandemic has further aggravated the psychological distress of health care professionals due to the double burden of high infection risk and the demands of dealing with a public health emergency [3]. A literature review and meta-analysis reported a prevalence range of 24.1% to 67.55% for anxiety, 12.1% to 55.89% for depression, and 29.8% to 62.99% for stress among health care professionals during the COVID-19 pandemic [4]. These numbers were much higher than those reported before the pandemic. For instance, Lv et al [5] surveyed 8028 Chinese health care professionals from 8 provinces of China and found that the rates of anxiety, depression, and sleep disorders were obviously higher during COVID-19 compared with before the pandemic.

A major driver of health care professionals’ psychological distress is burnout [6], a work-related stress syndrome resulting from continuous job pressures that individuals cannot effectively manage [7]. Burnout consists of 3 qualitative components: emotional exhaustion, depersonalization, and low personal accomplishment [7]. Health care professionals are at increased risk of burnout due to numerous stressful situations, such as witnessing the distress and death of patients; overloaded clinical work; time and role conflicts; and challenging interpersonal relationships with patients, colleagues, and leaders [8]. Approximately 1 in 3 physicians is experiencing burnout at any given time [9]. Burnout is estimated to have affected 10% to 70% of nurses and 30% to 50% of physicians and other health care professionals before COVID-19 [10]. A meta-analysis including 148 studies with 159,194 Asian health care professionals showed that the pooled prevalence for burnout was 68.3% during COVID-19 [11]. The positive link between burnout and psychological distress among health care professionals has been well established, with numerous studies showing that health care professionals with higher levels of burnout are also at increased risk of experiencing stress, depression, and anxiety [7,12,13].

Despite the high risk of burnout, not every health care professional experience severe psychological distress, which may be explained by the positive adaptation facing stress, such as psychological reflexibility. Psychological flexibility can be defined as “the ability to persist or to change behavior in a setting of competing psychological influences, guided by values and goals dependent on what the situation at hand affords” [14]. Psychological flexibility is an essential concept in the field of positive psychology and aligns well with the cognitive behavioral therapy framework [15]. A growing body of evidence suggests that psychological flexibility can mitigate the adverse impacts of burnout on psychological distress (ie, anxiety, depression, and stress) among health care professionals [16-18]. Therefore, psychological flexibility serves as a crucial psychological resilience factor against adverse mental health outcomes to improve health care professionals’ general well-being.

Health care professionals’ psychological distress and burnout may adversely affect the quality of care and patient safety, leading to increased medical errors and complaints, health care–associated infections, patient mortality, and decreased patient satisfaction [19]. On the system level, they may lead to low workforce productivity, poor work performance, high intention to leave, high turnover rate, and increased health care costs [20]. On a personal level, health care professionals with high levels of psychological distress and burnout are more likely to have risk behaviors such as substance abuse and even commit suicide [21]. Considering the high prevalence and severe outcomes of psychological distress and burnout among health care professionals, it is both urgent and critical to conduct effective interventions to address these problems among health care professionals.

Acceptance and commitment therapy (ACT) is a third-generation cognitive behavioral therapy aimed at fostering psychological flexibility through a range of mindfulness and values-based exercises while accepting difficult thoughts and feelings [22]. It involves 6 core therapeutic processes: acceptance, cognitive defusion, being present, self as context, values, and committed action [23]. ACT aims to enhance people’s psychological flexibility to help them accept and live their lives in a meaningful way instead of changing their life conditions [22]. ACT encourages people to maintain a mindful and accepting attitude, free themselves from mental constraints, and evaluate themselves situationally. It also helps them develop psychological flexibility, uncover their values, and form goals and action plans to achieve fundamental changes [23].

Ever since the first study on ACT was published [24], hundreds of randomized controlled trials (RCTs) have been conducted to test its effectiveness among various populations across various countries [25]. There is strong evidence base supporting the effectiveness of ACT as a transdiagnostic treatment in the clinical and general population with multiple conditions. Gloster et al [26] reviewed 20 meta-analyses of ACT effectiveness, reporting 100 controlled effect sizes across 12,477 participants grouped by target conditions and comparison group. They found that ACT was effective in various conditions, including anxiety, depression, substance use, pain, and transdiagnostic groups [26]. In addition, ACT demonstrated advantages over inactive controls, treatment as usual, and most active intervention conditions [26]. Furthermore, ACT has also been shown to be effective in improving the well-being of health care professionals. A recent meta-analysis of 10 RCTs revealed that ACT outperformed pooled control conditions in decreasing both general distress and work-related distress among health care professionals [27].

However, the application of ACT in health care professionals is limited due to the heavy workload and limited free time of health care professionals, making it challenging for them to attend face-to-face ACT interventions. These limitations could be addressed by internet-delivered ACT (iACT), which has gained increasing popularity due to its easy accessibility, greater convenience, and cost-effectiveness. Compared to face-to-face group interventions, iACT provides more convenience and reflexibility without the restriction of time, geography, or venue [28]. In addition, iACT offers greater privacy and can attract more health care professionals who have mental health problems but are reluctant to seek direct help due to their professional identities and concerns about privacy [29]. Furthermore, iACT is highly accessible due to the increasing number of internet users. Finally, iACT is less costly since it can be self-guided without relying heavily on psychotherapists as traditional face-to-face treatment [30]. Brown et al [31] conducted a systematic review and meta-analysis of the effectiveness of iACT. They found that iACT significantly decreased common mental disorders such as depression and anxiety and improved well-being [31]. In addition, they also observed high adherence rates, indicating the high acceptability of iACT in both patients and the public [31].

However, there is limited evidence of its effectiveness in improving health care professionals’ mental health in China. Thus, this study aims to examine the effectiveness of an iACT intervention program in reducing psychological distress (stress, anxiety, and depression) and burnout and improving psychological reflexibility among health care professionals in China.

## Methods

### Study Design and Participants

A parallel RCT with 4 repeated measures was conducted among health care professionals in Hunan province between October 2022 and February 2023. The study protocol was registered with the Chinese Clinical Trial Registry (ChiCTR2400093584).

The inclusion criteria of participants included (1) aged 18 years and older; (2) registered health care professionals with formal licenses issued by the National Health Department and with at least 1 year of working experience; and (3) able to use the internet and WeChat (Tencent). The exclusion criteria were (1) participants who were off duty due to sick leave, maternal leave, or other reasons during the study period; (2) participants who had a prior diagnosis of severe mental illness; (3) participants with severe suicidal ideation as assessed by the last item score of the Depression Anxiety and Stress Scales-21 (DASS-21)≥3 or suicidal behaviors; and (4) participants who had received oral psychotropic drug or psychotherapy treatment within the past three months. All participants provided informed consent before enrollment.

### Sample Size

The sample size was estimated using G*Power 3.1 (Heinrich-Heine-Universitat Dusseldorf, Dusseldorf, Germany). We selected “*F* test” and the test family “ANOVA: repeated measures, between factors” as the statistical test, and “DASS-21” as the key outcome variable when estimating the required sample size to achieve the minimal clinical difference. A previous meta-analysis showed that the average improvement effect of iACT intervention on depression and anxiety symptoms could be small, with an effect size of 0.24 to 0.38 and adherence rates between 53.33 and 97.33% [10]. The effect size was set at 0.24, α was set at .05, and the power was set at 0.80. Given that the surveys were to be repeated four times, assuming a repeated-measures correlation of 0.5 and an attrition rate of 22%, the required sample size was 107.

### Procedure

Potential participants were recruited through advertisements on WeChat, and those interested could scan a QR code to complete a web-based screening questionnaire to determine their eligibility. WeChat is a multifunctional messaging app that offers messaging, voice calls, video calls, mobile payments, miniprograms, and a wide range of other features for daily use [32]. Those who passed the screening criteria were assessed via telephone to confirm inclusion and exclusion criteria. Detailed information on the study's purpose, procedure, benefits, and risks was provided to all participants. After providing electronic informed consent, the participants were randomly allocated 1:1 to the iACT group or the waitlist control (WLC) group. A simple randomization method was used to randomize the participants. Researchers who were blinded to the study generated random sequences on a website and assigned participants to the appropriate group. In addition, data collectors were unaware of the group assignments throughout the study period.

All assessments were conducted through questionnaires on the Sojump website [33]. Sojump, also named Wenjuanxing in Chinese, is one of the largest web-based survey platforms in China [34]. It provides multiple free functions related to surveys, such as questionnaire development and distribution, data analysis, and result reporting, similar to Qualtrics or SurveyMonkey in the United States [34]. Four questionnaires were administered at the following 4 time points: baseline (T0), 2 weeks into the program (T1: week 2), right after the intervention (T2: week 6), and 4 weeks after the intervention (T3: week 10). Participants were compensated with a gift (approximately US $6) if they completed all 4 assessments.

### Ethical Considerations

The study protocol was approved by the ethics committees of Changsha Center for Disease Control and Prevention (2021003). Participants were fully informed of the study’s objective, procedures, benefits, and potential risks. They were guaranteed privacy and anonymity when participating in the study. All information and data were used for research purposes and would not be shared with a third party. Participation in the study was totally voluntary, and participants could withdraw from the study at any time, which would not affect their work and life. All participants provided electronic informed consent before participation. Participants were compensated with a gift (approximately US $6) if they completed all 4 assessments.

### Intervention

The intervention was adapted from Hayes’ [35] protocols, and the content was based on the 6 core processes of ACT: acceptance, cognitive defusion, being present, self as context, values, and committed action [36]. The components (ie, dosage, frequency, and duration) of the iACT were determined based on an extensive literature review and consultation with experts in iACT. Previous studies have shown that interventions with more sessions can produce more significant and long-lasting positive effects, presenting a dose-response relationship [37]. Therefore, we designed 28 sessions to maximize the intervention effects. Considering the busy clinical work of health care professionals, we limited each intervention session duration to 15-20 minutes to facilitate fragmented self-learning for health care professionals and minimize their participation burden. The intervention was tested among the general population in the early stages, and positive feedback was received.

The 6-week iACT intervention was offered through the Little Program on WeChat, and participants need to log in to the Little Program before using it. The flowchart of the specific intervention module and screenshots of the WeChat Mini Program are shown in Multimedia Appendix 1. The iACT program contained 28 sessions. The first 21 self-guided sessions were recorded by a psychologist and a meditation instructor, with each session unlocked every other day. Each session included a 10-15-minute recorded teaching video of a therapist, a 5-8-minute audio recording of guided mindfulness practice, and a journal writing task. The journals can be public or private between the “instructor” and the “student.” Participants received a WeChat message on the morning of practice day about the assigned session and practice assignment, which needed to be completed within 48 hours. After watching the video, listening to the audio, and submitting the journal, the participants were considered to have completed 1 session. After that, 7 web-based live conferences were held on days 6, 12, 18, 24, 30, 36, and 42 by an ACT therapist to review lessons, answer questions, and discuss difficult experiences. The number of sessions completed by participants is recorded in the background. Details about the sessions are shown in Table 1.

**Table 1:** Session themes.

Day	Self-guided session themes				Live conferences
	iACT^a^ teaching video	Mindfulness practices audio	Journal writing		
1	Understanding human suffering and the meaning of suffering	Understanding mindfulness	What might be holding you back from living the life you want?		
3	Search for the source of pain: our perspective on those difficulties	Serenity of the sea	What are your sensations and thoughts?”		
5	Escaping pain causes problems	Mountain meditation	Write down your coping strategies and their effects		
6	N/A^b^	N/A	N/A	✓	
7	Brain mechanism of avoidance	What took you away	Practice self-caring behavior		
9	Acceptance: facing our painful emotions, thoughts, feelings, and impulses with an open attitude	Back to anchor	Make a list of your emotional events: “I (experienced)... I feel...”		
11	Cultivate a positive attitude	Naming Emotions	Positive mindset toolkit		
12	N/A	N/A	N/A	✓	
13	Get out of your mind and observe your thoughts	Finding inherent patterns	What are you thinking about right now?		
15	Introduce cognitive fusion	Observe the thinking train	What are your emotions, sensations, and thoughts?		
17	Introduce cognitive defusion and defusion techniques	Leaves on running water	What were my emotions, sensations, and thoughts during this meditation?		
18	N/A	N/A	N/A	✓	
19	The 3 senses of self	Metta Meditation	Retell your story		
21	Being the observing self	Deepening of Metta meditation: self-care	Describe your sensation, experience, and realization of the practice		
23	Introduce mindfulness	Shelter of mercy	Pay attention to 5 things		
24	N/A	N/A	N/A	✓	
25	Experience mindfulness practice	Mindfulness body scan	Your sensation and experience		
27	Sitting meditation	Sitting meditation	Your experience		
29	Practice mindful eating	Practice eating a raisin mindfully	What do you feel?		
30	N/A	N/A	N/A	✓	
31	Values as chosen life directions	Let go of your worries	Imagine you are at your 80th birthday party and your friend is giving a speech. Write down what you hear.		
33	What values are and are not	Compassion and value	10 areas of value		
35	Choose your values	Lake and pebbles—value discovery	Rank and test your value		
36	N/A	N/A	N/A	✓	
37	Make an action plan	Accurately identify risks and resources	Target exercise table		
39	Build patterns of effective action	Fulfillment Exercises—empower action	Write down the new behavior patterns and action plans you want to create		
41	Applying values to daily life	Realize value through action	What thoughts come to your mind during a mindfulness practice?		
42	N/A	N/A	N/A	✓	

^a^iACT: internet-based acceptance and commitment therapy.

^b^N/A: not applicable.

### Interventionists

The interventionists included a psychologist, who was a psychotherapy supervisor with extensive experience in ACT; a meditation instructor, who was also an ACT therapist; and an ACT therapist. The psychologist and meditation instructor were responsible for recording the first 21 self-guided sessions since these sessions included highly standardized teaching modules that required high qualifications. In contrast, the ACT therapist was in charge of the last 7 web-based live conferences, which required less qualification but more flexibility in coordinating and responding to various needs. All interventionists received standard training in ACT and had rich experience in delivering iACT. They provided the intervention following a highly standardized treatment protocol and were supervised during the intervention.

### WLC Group

Participants in the WLC group received no intervention but could access other forms of care at their freedom, the same way as the intervention group. They were instructed that they could seek formal and informal help whenever they encountered psychological problems during the study, as they normally would. Four weeks after the study completion, the WLC group could start the iACT of their choice.

### Measures

#### Demographic Information

Demographic information such as age, sex, marital status, and education was collected at baseline.

#### Primary Outcome Measure

The DASS-21 [38] was used to assess health care professionals’ psychological distress with 3 subscales: depression, anxiety, and stress. The DASS-21 contains 21 items (7 items in each subscale), and each item is rated on a 4-point Likert-type scale ranging from 0=did not apply to me at all to 3=applied to me very much or most of the time. The total score ranges from 0 to 63, with a higher score indicating higher psychological distress. The DASS-21 has shown good internal consistency and convergent and discriminant validity in Chinese samples [39,40]. In this study, the DASS-21 demonstrated good internal consistency with a Cronbach α of 0.91 for the total scale, 0.87 for the depression subscale, 0.78 for the anxiety subscale, and 0.80 for the stress subscale.

#### Secondary Outcome Measure

Burnout was measured by the Maslach Burnout Inventory-General Survey (MBI-GS) developed by Maslach et al [41] and adapted by Li and Shi [42]. The Chinese version of MBI-GS [43] contains 15 items under 3 dimensions: exhaustion (5 items), cynicism (4 items), and reduced personal accomplishment (6 items, reverse scored). Each item is rated on a 7-point scale ranging from 0 to 6 according to the frequency of symptoms. The weighted sum score = emotional exhaustion score×0.4+ cynicism score×0.3+ reduced personal accomplishment×0.3, with a cutoff of 1.5 distinguishing between those with (≥1.5) and those without burnout (<1.5). In our study, the MBI-GS demonstrated good internal consistency with a Cronbach α of 0.833 for the total scale.

Psychological flexibility was assessed using the Comprehensive Assessment of ACT Processes (CompACT) as a process measurement for ACT. The CompACT [44] consists of 23 items under 3 subscales: openness to experience (10 items), behavioral awareness (5 items), and valued action (8 items). Each item is rated on a 7-point Likert scale from 0 to 6. The total score ranges from 0 to 138, with a lower total score indicating greater psychological flexibility. The CompACT demonstrated good internal consistency in this study with a Cronbach α of 0.893.

### Data Analysis

All data were analyzed via IBM SPSS statistics (version 20.0). Continuous variables were expressed as means and SDs, while categorical variables were presented as numbers and proportions. The independent samples 2-tailed *t* tests and chi-square tests were conducted to assess the homogeneity of the pretest data between the intervention and control groups. Generalized estimating equations (GEEs) were used to compare the outcomes (DASS-21, MBI-GS, and CompACT score changes) between the 2 groups and assess the group, time, and group-by-time interaction effects. Assessments conducted at baseline (T0), week 2 (T1), week 6 (T2), and week 10 (T3) were included as outcomes in the GEE analysis, with 0.2, 0.5, and 0.8 representing a small, medium, and large effect, respectively. The effect size was conducted through a web-based effect size calculator [45]. A subgroup analysis was performed using the independent samples 2-tailed *t* test to explore the sex differences in the intervention effects. In order to test whether participants with more severe psychological distress benefit more from the iACT program, we conducted a sensitive analysis on participants with a DASS-21 score≥20 (65th percentile) at baseline. The significance level was set at *P*=.05; all tests were 2-tailed.

## Results

### Participant Recruitment and Baseline Characteristics

**Figure 1:**
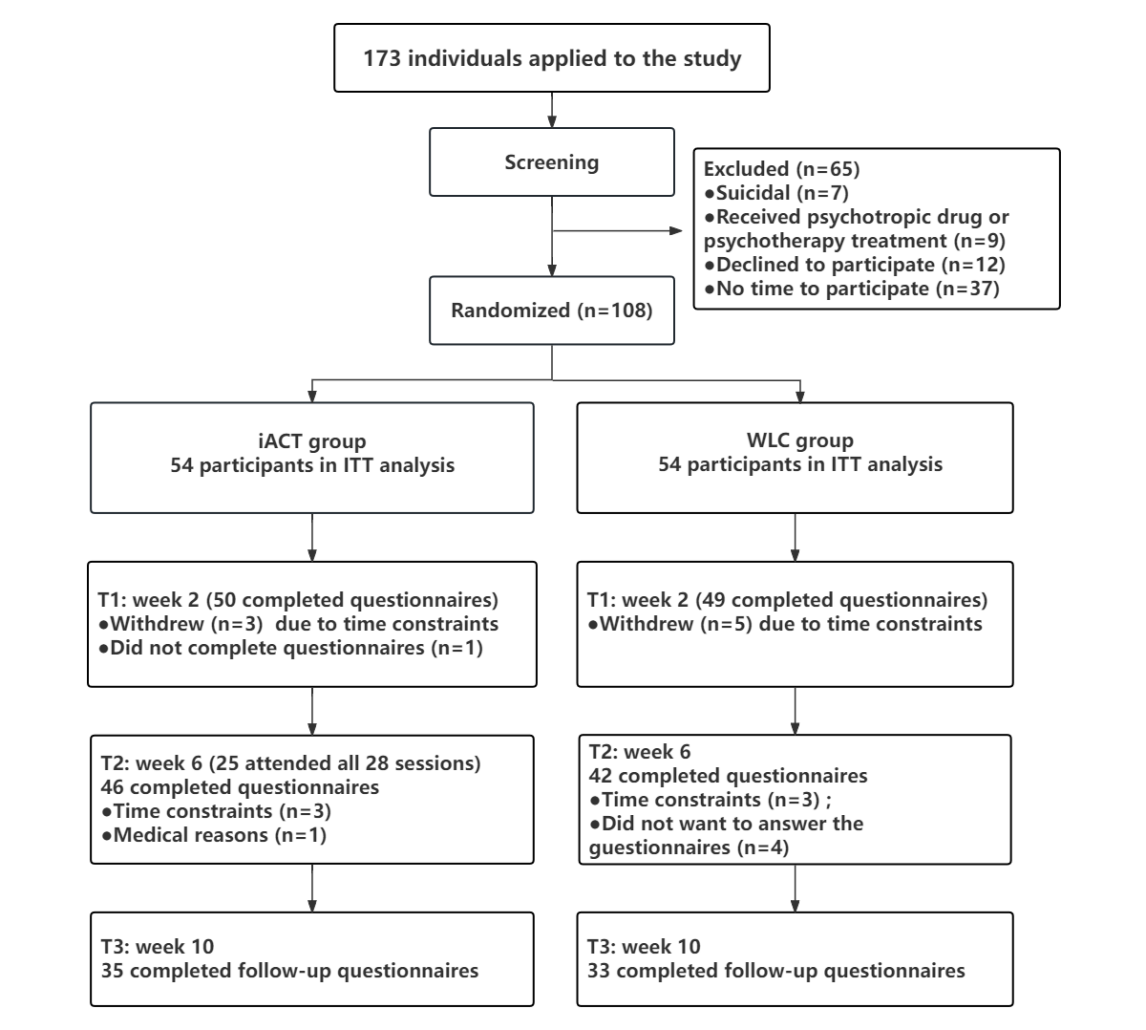
CONSORT (Consolidated Standards of Reporting Trials) flow diagram of participant recruitment and retention in the intervention. iACT: internet-based acceptance and commitment therapy; ITT: intention-to-treat; WLC: waitlist control group.

A total of 173 health care professionals were recruited, among whom 108 (n=42, 38.9% physicians; n=58, 53.7%, nurses; and n=8, 7.4%, support staff) were included and randomized into the iACT group (n=54) and the WLC group (n=54). Among the 108 participants, 99 (91.7%) completed at least 1 assessment, and 68 (63%) completed the follow-up assessment at week 10, including 35 in the iACT group and 33 in the WLC group (Figure 1). Among the final 68 participants, there were 52 (76.5%) female participants and 16 (23.5%) male participants. There were no significant differences in the attrition rates between the intervention group and the control group (*χ*^2^_1_=0.159; *P*=.690). Of the 54 participants in the iACT group, all attended at least 2 sessions (including self-guided sessions and the live sessions), 25 attended all 28 sessions, and 44 attended more than half of the total sessions. On average, participants attended 20 (71%) of the total sessions.

At baseline, the participants had a mean age of 38.45 (SD 7.26) years, and 81.5% (88/108) were female. Their mean scores for depression, anxiety, stress, burnout, and psychological flexibility were 5.56 (SD 3.53), 4.78 (SD 3.10), 7.43 (SD 3.29), 2.49 (SD 0.77), and 31.34 (SD 9.08), respectively (Table 1). There were no significant differences in baseline characteristics between the intervention group and the control group (Table 2) and between the completed sample and the missing sample (Multimedia Appendix 2).

**Table 2:** Baseline characteristics of participants.

Variables			Total sample (n=108)		iACT^a^ group (n=54)		WLC^b^ group (n=54)
**Age (years), mean (SD)**			38.45 (7.26)		38.35 (6.85)		38.56 (7.70)
**Sex, n (%)**							
	Male	20 (18.5)		11 (20.4)		9 (16.7)	
	Female	88 (81.5)		43 (79.6)		45 (83.3)	
**Marital status, n (%)**							
	Single	13 (12.1)		8 (14.8)		5 (9.3)	
	Married	90 (83.3)		44 (81.5)		46 (85.2)	
	Others	5 (4.6)		2 (3.7)		3 (5.6)	
**Occupational role, n (%)**							
	Physician	37 (34.3)		21 (38.9)		19 (35.2)	
	Nurse	64 (59.3)		26 (48.1)		32 (59.3)	
	Other	7 (6.5%)		7 (13.0%)		3 (5.6%)	
**Education, n (%)**							
	Senior high school	3 (2.8%)		1 (1.9%)		2 (3.7%)	
	College degree or higher	105 (97.2%)		53 (98.1%)		52 (96.3%)	
**DASS-21^c^, mean (SD)**			17.76 (8.56)		17.24 (9.85)		18.28 (7.10)
	Depression	5.56 (3.53)		5.30 (3.99)		5.81 (3.01)	
	Anxiety	4.78 (3.10)		4.74 (3.37)		4.81 (2.84)	
	Stress	7.43 (3.29)		7.20 (3.66)		7.65 (2.89)	
**MBI-GS**^d^, **mean (SD)**			2.49 (0.77)		2.43 (0.82)		2.56 (0.71)
**CompACT**^e^, **mean (SD)**			31.34 (9.08)		32.02 (9.20)		30.67 (9.00)
	Openness to experience	11.10 (5.51)		11.04 (5.63)		11.17 (5.43)	
	Behavioral awareness	9.41 (5.16)		9.89 (5.16)		8.93 (5.15)	
	Valued action	10.83 (5.15)		11.09 (5.53)		10.57 (4.77)	

^a^iACT: internet-based acceptance and commitment therapy.

^b^WLC: waitlist control.

^c^DASS-21: Depression Anxiety and Stress Scales-21.

^d^MBI-GS: Maslach Burnout Inventory-General Survey.

^e^CompACT: Comprehensive Assessment of ACT Processes.

### Primary Outcomes

As shown in Table 3, the GEE model revealed a statistically significant group effect (*P*<.001) and group-by-time interaction effect (*P*=.04) on the DASS-21 score. The iACT group showed more substantial changes in DASS-21 score at weeks 2, 6, and 10 (β=–3.85, –6.36, –7.50, respectively) than the WLC group. At weeks 2, 6, and 10, the iACT group consistently showed significantly lower scores in the subscales of depression (β=–1.24, –2.42, –2.87, respectively), anxiety (β=–1.25, –2.00, –2.12, respectively) and stress (β=–1.42, –1.98, –2.59, respectively) than the WLC group (all *P*<.05). The intergroup effect sizes for psychological distress at week 6 and week 10 were 0.82 (95% CI 0.39-1.26) and 1.08 (95% CI 0.57-1.59), respectively.

**Table 3:** Between-group comparisons of changes in outcome variables over time.

Outcome and time			β (95% CI)		*P* value		Cohen *d* (95% CI)		Group effect						Time effect					Group × time effect				
									Wald chi-square (*df*)			*P* value			Wald chi-square (*df*)		*P* value			Wald chi-square (*df*)			*P* value	
**DASS-21^a^**										40.29 (1)			<.001^b^		0.298 (2)			.86			6.622 (2)			.04^b^
	T0^c^	–1.04 (–4.31 to 2.24)		.53		Reference^d^																		
	T1^e^	–3.85 (–5.82 to –1.88)		<.001^b^		0.65 (0.24-1.05)																		
	T2^f^	–6.36 (–9.10 to –3.62)		<.001^b^		0.82 (0.39-1.26)																		
	T3^g^	–7.50 (–10.18 to –4.82)		<.001^b^		1.08 (0.57-1.59)																		
**Depression**										35.66 (1)			<.001^b^		0.593 (2)			.74			11.43 (2)			.003^b^
	T0	–0.52 (–1.87 to 0.83)		.22		Reference																		
	T1	–1.24 (–2.08 to –0.40)		.004^b^		0.45 (0.05-0.85)																		
	T2	–2.42 (–3.34 to –1.50)		<.001^b^		0.84 (0.40-1.28)																		
	T3	–2.87 (–3.81 to –1.92)		<.001^b^		1.03 (0.52-1.53)																		
**Anxiety**										31.96 (1)			<.001^b^		0.67 (2)			.71			3.18 (2)			.20
	T0	–0.07 (–1.26 to 1.12)		.60		Reference																		
	T1	–1.25 (–1.87 to –0.63)		<.001^b^		0.68 (0.28-1.09)																		
	T2	–2.00 (–2.89 to –1.10)		<.001^b^		0.77 (0.34-1.20)																		
	T3	–2.12 (–3.11 to –1.14)		<.001^b^		0.85 (0.35-1.35)																		
**Stress**										24.75 (1)			<.001		5.16 (2)			.08			4.14 (2)			.13
	T0	–0.44 (–1.70 to 0.81)		0.46		Reference																		
	T1	–1.42 (–2.26 to –0.58)		.001^b^		0.54 (0.14-0.94)																		
	T2	–1.98 (–3.24 to –0.72)		.002^b^		0.55 (0.12-0.98)																		
	T3	–2.59 (–3.65 to –1.53)		<.001^b^		0.93 (0.43-1.44)																		
**MBI-GS^h^**										63.49 (1)			<.001^b^		26.29 (2)			<.001^b^			19.84 (2)			<.001^b^
	T0	–0.13 (–0.42 to 0.16)		.38		Reference																		
	T1	–0.53 (–0.80 to –0.27)		<.001^b^		0.57 (0.17-0.97)																		
	T2	–0.92 (–1.12 to –0.71)		<.001^b^		1.42 (0.95-1.89)																		
	T3	–1.08 (–1.33 to –0.83)		<.001^b^		1.52 (0.98-2.06)																		
**CompACT^i^**										40.95 (1)			<.001^b^		34.57 (2)			<.001^b^			15.8 (2)			<.001^b^
	T0	1.35 (–2.12 to 4.82)		0.44		Reference																		
	T1	–3.64 (–5.80 to –1.48)		.001^b^		0.69 (0.28-1.09)																		
	T2	–7.64 (–9.98 to –5.31)		<.001^b^		1.23 (0.77-1.69)																		
	T3	–8.18 (–10.88 to –5.49)		<.001^b^		1.15 (0.64-1.66)																		
**Openness to experience**										6.94 (1)			.008^b^		21.86 (2)			<.001^b^			6.84 (2)			.03^b^
	T0	–0.13 (–2.24 to 1.98)		.72		Reference																		
	T1	–0.81 (–2.17 to 0.56)		.248		0.21 (–0.18 to 0.61)																		
	T2	–2.16 (–3.55 to –0.77)		.002^b^		0.49 (0.06-0.91)																		
	T3	–2.20 (–3.75 to –0.65)		.005^b^		0.44 (–0.04 to 0.92)																		
**Behavioral awareness**										5.67 (1)			0.02^b^		13.33 (2)			.001^b^			6.97 (2)			.03^b^
	T0	0.96 (–1.00 to 2.93)		.30		Reference																		
	T1	–0.23 (–1.65 to 1.18)		.749		0.24 (–0.16 to 0.63)																		
	T2	–1.79 (–2.99 to –0.60)		.003^b^		0.70 (0.27-1.13)																		
	T3	–2.07 (–3.48 to –0.66)		.004^b^		0.69 (0.20-1.18)																		
**Valued action**										30.39 (1)			<.001^b^		15.48 (2)			<.001^b^			8.66 (2)			.01^b^
	T0	0.52 (–1.45 to 2.49)		.74		Reference																		
	T1	–2.54 (–3.81 to –1.27)		<.001^b^		0.66 (0.26-1.07)																		
	T2	–3.58 (–4.81 to –2.35)		<.001^b^		0.92 (0.48-1.36)																		
	T3	–3.81 (–5.15 to –2.47)		<.001^b^		0.88 (0.38-1.38)																		

^a^DASS-21: Depression Anxiety and Stress Scales-21.

^b^The *P* value was statistically significant.

^c^T0: baseline.

^d^Effect size of the intervention was 0.

^e^T1: week 2.

^f^T2: week 6.

^g^T3: week 10.

^h^MBI-GS: Maslach Burnout Inventory-General Survey.

^i^CompACT: Comprehensive Assessment of ACT Processes.

### Secondary Outcomes

Table 3 also showed statistically significant group effect, time effect, and group-by-time interaction effects on burnout and psychological flexibility (all *P*<.05). Compared to the WLC group, the iACT group had significantly greater changes in burnout score from T0 to T3 (β=–1.08), from T0 to T2 (β=–.92), and from T0 to T1 (β=–.57). The intergroup effect sizes for burnout at week 6 and week 10 were 1.42 (95% CI 0.95-1.89) and 1.52 (95% CI 0.98-2.06), respectively. The ACT group showed more substantial changes in CompACT score at weeks 2, 6, and 10 (β=–3.64, –7.64, –8.18, respectively) than the WLC group, also reflected in its 3 subscales of openness to experience, behavioral awareness, and valued action. The intergroup effect sizes for psychological flexibility at week 6 and week 10 were 1.23 (95% CI 0.77-1.69) and 1.15 (95% CI 0.64-1.66), respectively.

### Subgroup Analysis

Considering the large proportion of female individuals in the final sample, we further conducted a sex-based subgroup analysis to test if there were any significant sex differences in the intervention effects. Table 4 shows the results of the independent samples 2-tailed *t* tests to assess the primary and secondary outcomes. There were no statistical sex differences in all outcomes, including the DASS-21, MBI-GS, and CompACT scores (all *P*>.05).

**Table 4:** Subgroup analysis of the primary and secondary outcomes at week 10 (n=35).

	Male (n=9), mean (SD)	Female (n=26), mean (SD)	*t* test (*df*)	*P* value
DASS-21^a^ (T3^b^-T0^c^)	–2.33 (10.51)	–6.04 (7.90)	1.11 (33)	.27
MBI-GS^d^ (T3-T0)	–1.19 (0.66)	–0.87 (0.83)	–1.05 (33)	.30
CompACT^e^ (T3-T0)	–7.11 (4.26)	–9.19 (10.61)	0.83 (32)	.42

^c^DASS-21: Depression Anxiety and Stress Scales-21.

^b^T3: week 10.

^c^T0: baseline.

^d^MBI-GS: Maslach Burnout Inventory-General Survey.

^e^CompACT: Comprehensive Assessment of ACT Processes.

### Sensitivity Analysis

To examine the effect of the interventions on participants who exhibited higher levels of distress, we conducted a sensitivity analysis on the participants with a DASS-21 score ≥20 (65th percentile) at baseline (21 in the iACT group and 20 in the WLC group). As shown in Table 5, the scores of the DASS-21 total scale and its 3 subscales increased in the iACT group but decreased in the WLC group. The iACT group showed more substantial changes in DASS-21 score at weeks 2, 6, and 10 (β=–7.91, –8.44, –11.11, respectively) than the WLC group. At follow-up, the ACT group had more participants who no longer met the DASS-21 criterion for significant distress than the WLC group (77% vs 64%). Regarding burnout, compared to the WLC group, the iACT group showed more remarkable changes in burnout scores from T0 to T3 (β=–0.74), from T0 to T2 (β=–0.74), and from T0 to T1 (β=–0.48). As for psychological flexibility, the ACT group showed more substantial changes in CompACT score at weeks 2, 6, and 10 (β=–6.41, –9.35, –8.30, respectively), also reflected in its 3 dimensions of openness to experience, behavioral awareness, and valued action.

**Table 5:** Changes in outcome variables over time for participants with DASS-21 total scores ≥20 at baseline (n=41).

Outcome and time		β (95% CI)	*P* value	Cohen *d* (95% CI)	Group effect				Time effect				Group × time effect			
					Wald chi-square (*df*)		*P* value		Wald chi-square (*df*)		*P* value		Wald chi-square (*df*)		*P* value	
**DASS-21^a^**						39.72 (1)		<.001^b^		0.03 (2)		.99		1.52 (2)		.47
	T0^c^	1.00 (–2.86 to 4.86)	.66	Reference^d^												
	T1^e^	–7.91 (–10.79 to –5.02)	<.001^b^	1.64 (0.91-2.38)												
	T2^f^	–8.44 (–13.68 to –3.19)	.002^b^	0.83 (0.09-1.57)												
	T3^g^	–11.11 (–16.41 to –5.81)	<.001^b^	1.59 (0.55-2.64)												
**Depression**						32.63 (1)		<.001^b^		0.36 (2)		.84		2.16 (2)		.34
	T0	0.36 (–1.67 to 2.39)	.72	Reference												
	T1	–2.37 (–3.63 to –1.11)	<.001^b^	1.02 (0.34-1.70)												
	T2	–2.84 (–4.28 to –1.40)	<.001^b^	0.98 (0.22-1.73)												
	T3	–3.49 (–5.24 to –1.75)	<.001^b^	1.25 (0.25-2.24)												
**Anxiety**						41.66 (1)		<.001^b^		0.36 (2)		.83		1.17 (2)		.56
	T0	0.12 (–1.67 to 1.90)	.47	Reference												
	T1	–2.66 (–3.63 to –1.68)	<.001^b^	0.79 (0.005-1.53)												
	T2	–2.92 (–4.72 to –1.12)	.001^b^	1.53 (0.50-2.57)												
	T3	–3.86 (–5.73 to –1.98)	<.001^b^	1.20 (0.21-2.18)												
**Stress**						20.09 (1)		<.001^b^		0.74 (2)		.69		1.66 (2)		.44
	T0	0.52 (–1.23 to 2.28)	.49	Reference												
	T1	–2.79 (–4.24 to –1.35)	<.001^b^	1.10 (0.42-1.78)												
	T2	–2.66 (–5.14 to –0.17)	.04	0.50 (–0.23 to 1.22)												
	T3	–4.11 (–6.25 to –1.97)	<.001^b^	1.45 (0.43-2.47)												
**MBI-GS^h^**						15.68 (1)		<.001^b^		8.22 (2)		.02^b^		2.47 (2)		.29
	T0	–0.12 (–0.59 to 0.34)	.59	Reference												
	T1	–0.48 (–0.91 to –0.05)	.03^b^	0.44 (–0.21 to 1.08)												
	T2	–0.74 (–1.07 to –0.42)	<.001^b^	1.18 (0.41-1.95)												
	T3	–0.74 (–1.16 to –0.32)	.001^b^	1.12 (0.14-2.10)												
**CompACT^i^**						27.15 (1)		<.001^b^		7.40 (2)		.03^b^		5.04 (2)		.08
	T0	2.12 (–3.07 to 7.31)	.41	Reference												
	T1	–6.41 (–9.52 to –3.29)	<.001^b^	1.25 (0.55-1.94)												
	T2	–9.35 (–12.62 to –6.07)	<.001^b^	1.58 (0.77-2.39)												
	T3	–8.30 (–12.54 to –4.06)	<.001^b^	1.29 (0.29-2.29)												
**Openness to experience**						9.85 (1)		.002^b^		6.67 (2)		.04^b^		9.20 (2)		.01^b^
	T0	0.13 (–2.0 to 2.26)	.82	Reference												
	T1	–2.56 (–4.60 to 0.52)	.01^b^	0.74 (0.08-1.39)												
	T2	–4.23 (–6.26 to –2.19)	<.001^b^	0.99 (0.24-1.75)												
	T3	–3.20 (–5.85 to –0.56)	.02^b^	0.70 (–0.24 to 1.64)												
**Behavioral awareness**						9.32 (1)		.002^b^		3.11 (2)		.21		0.53 (2)		.77
	T0	–0.15 (–3.00 to 2.71)	.92	Reference												
	T1	–1.73 (–3.85 to 0.38)	.11	0.44 (–0.20 to 1.09)												
	T2	–2.27 (–3.88 to –0.65)	.006^b^	0.77 (0.03-1.51)												
	T3	–2.61 (–4.42 to –0.79)	.005^b^	1.16 (0.17-2.14)												
**Valued action**						16.46 (1)		<.001^b^		1.64 (2)		.44		1.61 (2)		.45
	T0	2.14 (–0.21 to 4.50)	.07	Reference												
	T1	–1.57 (–2.78 to –0.36)	.01^b^	0.95 (0.28-1.62)												
	T2	–2.31 (–3.45 to –1.17)	<.001^b^	1.21 (0.43-1.98)												
	T3	–2.29 (–3.71 to –0.87)	.002^b^	0.82 (–0.13 to 1.76)												

^a^DASS-21: Depression Anxiety and Stress Scales-21.

^b^The *P* value was statistically significant.

^c^T0: baseline.

^d^Effect size of the intervention was 0.

^e^T1: week 2.

^f^T2: week 6.

^g^T3: week 10.

^h^MBI-GS: Maslach Burnout Inventory-General Survey.

^i^CompACT: Comprehensive Assessment of Acceptance and Commitment Therapy Processes.

## Discussion

### Principal Findings

Our results demonstrated the effectiveness of the iACT intervention in reducing health care professionals’ burnout and psychological distress, including stress, anxiety, and depression, as well as improving their psychological flexibility. The effect was more substantial among health care professionals with higher baseline psychological distress. Following 6 weeks of iACT intervention, the iACT group had more significant decreases in psychological distress and burnout and a more significant increase in psychological flexibility than the WLC group. The effects persisted at follow-up assessments 1 month later. These results are consistent with a large amount of evidence demonstrating the efficacy of iACT in individuals with different psychological conditions [46-49]. A meta-analysis found that iACT demonstrated small yet significant and enduring effects on depression and anxiety compared to control groups [50].

The level of psychological distress among health care professionals in our study was comparable to that reported in previous studies in China using the same assessment tool [51]. Deng et al [51] conducted a meta-analysis including 34 studies to evaluate and compare the prevalence of psychological distress among health care professionals and the general public before and after COVID-19. Among health care professionals, the prevalence of depression and anxiety was 40% and 38% before COVID-19, 31% and 40% during COVID-19, and decreased to 22% and 22% after COVID-19, respectively [51]. Among the general public, the prevalence of depression and anxiety was 33% and 24% before COVID-19, 26% and 22% during COVID-19, and increased to 62% and 44% after COVID-19, respectively [51]. These findings suggested that the COVID-19 epidemic affected health care professionals more severely than the public. However, health care professionals tend to recover more quickly after the pandemic, possibly due to better professional knowledge, cognition, experience, and preparation in response to the pandemic [52].

The DASS-21 total score of the iACT group at baseline in our study was similar to that of undergraduate first-year students in the Levin RCT study [53]. However, the change in the DASS-21 total score from baseline to post-assessment (–6.36) in our study was more significant than in the Levin study (–4.59). One explanation may be related to our study’s longer duration and more courses. The intervention program in our research spanned 6 weeks and consisted of 28 sessions, while the Levin program consisted of 2 web-based multimedia lessons and supplementary tailored emails. Most previous studies had a duration of intervention between 3 and 13 weeks. A meta-analysis including 10 RCT studies indicated that studies with more treatment sessions were associated with larger effect sizes for psychological distress [27].

In general, our study showed positive effects of iACT in alleviating depressive symptoms, which is consistent with the bulk of previous evidence. For instance, Lu et al [54] conducted an RCT and randomly assigned 145 nurses to either the 5-week fully automated intervention or the waiting group. The intervention group showed significant improvement in anxiety and depressive symptoms, which sustained even 3 months after the intervention [54]. Similar positive findings have also been reported by other studies, which consistently showed robust evidence to support the efficacy of iACT in benefiting individuals with depressive symptoms [36,55-58]. Thompson et al [36] conducted a systematic review and meta-analysis across 25 studies and found that iACT was effective in improving all outcomes at post-assessment and maintained at follow-up across diverse populations.

Our study showed that the iACT intervention significantly improved psychological flexibility, which was reflected in the more substantial decreases in the CompACT scale and its three subscales in the iACT group than in the WLC group. The low level of psychological flexibility is a significant risk factor for many psychological and behavioral problems [59]. Previous research found that psychological flexibility mediated or partially mediated the improvement of psychopathological symptoms such as anxiety and depression through ACT intervention [54,60,61]. For instance, Zhao et al [61] conducted an RCT and randomly assigned 124 Chinese university students to either the iACT intervention consisting of 6 weekly 30-minute units or the WLC group. They found that psychological flexibility significantly mediated the associations between iACT and both depressive symptoms and positive mental health [61]. These findings are in line with the framework of ACT, which focuses on using multiple therapeutic methods to improve psychological flexibility as a means to improve well-being. Psychological flexibility includes 3 overlapping subcomponents: openness (the willingness to have unwanted thoughts and feelings), awareness (the ability to notice one’s experiences mindfully), and engagement (actions that enable progress on one’s goals and values) [62]. All these subcomponents showed significant improvement after the iACT intervention, indicating their similar and equally important roles in mediating the intervention effects.

Noteworthily, we observed growing intervention effects with each subsequent week, which persisted even 4 weeks after the intervention, indicating the sustainability of the improvement effects. The improved effects may be explained by the dose-response relationship between the intervention and the effects and the time-accumulating effects [37]. As the intervention goes on, its health-promoting effects also accumulate over time, leading to persistent improvement that may last for a certain period of time after the intervention. However, the health benefits of the intervention may not last over an extended period after the intervention is terminated, raising concerns about sustainability [63]. The lack of sustainability is a common phenomenon in most intervention studies and a major contributor to the research-to-practice gap [64]. For instance, it has been estimated that it may take about 17 years to implement only 14% of evidence-based research outcomes in real-world settings [65]. Therefore, considering the temporal dynamics of the intervention effects, it is suggested that a long-term maintenance program after the iACT intervention may be necessary for the long-term well-being of health care professionals. Compared to the traditional offline intervention, iACT has the advantages of easy access, low cost, and flexible schedule without time or geography restrictions [54], which paves the way for the development of more affordable and cost-effective long-term maintenance booster programs in the future.

The iACT intervention program in our study combined self-management with therapist guidance. Despite the web-based nature of the intervention, participants attended 71% of sessions of the iACT programs; this is consistent with other studies showing a compliance rate ranging from 53.33% to 97.33% [66-68]. The adherence rate is a crucial indicator reflecting how well an intervention aligns with individuals' needs and expectations and achieves its intended benefits, especially in the context of the psychological well-being of health care professionals [54]. The high adherence rate observed in our study indicates the high feasibility, accessibility, and acceptability of iACT among health care professionals, as well as the positive experiences of health care professionals related to iACT [54]. It seems that interventions with therapist guidance had greater effectiveness in reducing psychological distress and burnout and improving psychological flexibility than non-guided iACT [50]. Further studies are needed to determine the optimal therapist involvement that balances efficacy and costs.

### Strengths and Limitations

Our study contributes to the literature by providing comprehensive and robust evidence on the effectiveness of iACT in decreasing stress, anxiety, depression, and burnout, as well as improving psychological flexibility among health care professionals in China. The study has several strengths, including a high adherence rate suggestive of high acceptance of the intervention by the participants, a long intervention duration with more treatment sessions to ensure a large effect size, and intensive follow-up evaluation with multiple time points encompassing pre-, mid-, and postintervention. Furthermore, the intervention is delivered through WeChat, the most commonly used social media in China. Health care professionals can easily access it without downloading any additional software, which can dramatically save time and energy for health care professionals who are already overwhelmed by heavy clinical workloads and limited free time. All these factors greatly enhance the effectiveness of the iACT intervention. In addition, we used multiple analysis strategies, including GEE, 2-tailed *t* tests, and chi-square tests, combined with subgroup analysis and sensitivity analysis to ensure the robustness of our results.

However, our findings should also be interpreted with caution due to several limitations. First, the sample was predominantly female, which may limit the generalization of the findings to males. However, subsequent sex-based subgroup analysis showed no significant differences. Future studies may consider recruiting a more sex-balanced sample to validate our results. Second, potential sampling bias may exist due to the self-selective nature of the web-based recruitment strategy. Health care professionals with higher levels of psychological distress who may benefit more from the intervention may be more likely to actively participate in the study, leading to more significant observed intervention effects. While large effect sizes were observed for decreases in general distress in participants and in those who exhibited higher levels of distress, only tentative conclusions can be made given the potential sampling bias. Third, all outcome variables were assessed subjectively based on self-report and are subject to bias such as recall bias and social desirability bias. Future studies may consider combining some objective measurement indicators to get a more accurate outcome assessment. Fourth, although there were no statistical differences in the baseline characteristics between participants who completed the intervention and those who dropped out, attrition may introduce bias and limit the generalizability of the findings. Future studies should explore multiple strategies to improve participant retention. Fifth, participants in this study were followed up for only 4 weeks after the intervention, which may limit the external validity of the results. More RCT studies with more extended follow-up periods are warranted to evaluate the long-term effectiveness of iACT in the future. Finally, we did not assess the interventionists' abilities and the intervention fidelity. However, the interventionists were highly qualified professionals with extensive experience in ACT, and they followed a highly standardized treatment protocol. Future studies should employ standard strategies, such as the recommendations from the Behavior Change Consortium established by The National Institutes of Health [69], to evaluate the intervention process and test the intervention fidelity.

### Challenges and Implications

While our study provides promising evidence of the effectiveness of iACT in improving the well-being of health care professionals, challenges may exist in scaling up this program to the real-world practice of hospitals and health care professionals. It has been widely acknowledged that interventions that have been shown effective in research settings may not equally be applied and widely adopted in practice and community-based settings, also known as the research-to-practice gap [70]. Contextual barriers (such as lack of supporting policies and regulations), health system barriers (such as the hospital’s lack of administrative support and resources to build up and maintain the program), and individual barriers (such as health care professionals’ lack of time, interest, and motivation in engaging in the program) may hinder the widespread adoption of the program [71].

Therefore, to address the research-to-practice gap and improve the adoption of the iACT program among health care professionals, we need to overcome those barriers using a multilevel approach. For instance, organizational governance should be strengthened to promote awareness of the iACT in health care organizations, and financial and physical resources should be assigned to these organizations to facilitate the implementation of the iACT. For health care professionals, it is necessary to conduct specialized education and training to help them better understand the advantages of iACT and enable them to be better consumers and users of the program. In addition, cultivating scholar-practitioners, who are both producers and consumers of the intervention, and promoting practice-based research are also promising approaches to integrating research and practice to ensure the wide dissemination and adoption of the iACT among health care professionals [64].

### Conclusions

In conclusion, this study adds empirical evidence of the efficacy of iACT training in decreasing health care professionals’ psychological distress and burnout and improving their psychological flexibility. Our findings provide implications and guidance for the development and wide implementation of iACT in health care settings to improve the mental health of health care professionals.

## Appendix

Multimedia Appendix 1 the WeChat Mini Program.

Multimedia Appendix 2 Comparison of baseline general information for completed and missed samples.

Multimedia Appendix 3 CONSORT-EHEALTH checklist for RCTs.

## Abbreviations

ACT: acceptance and commitment therapy
CompACT: Comprehensive Assessment of Acceptance and Commitment Therapy Processes
DASS-21: Depression Anxiety and Stress Scales-21
GEE: generalized estimating equation
iACT: internet-based acceptance and commitment therapy
MBI-GS: Maslach Burnout Inventory-General Survey
RCT: randomized controlled trial
WLC: waitlist control

## Data Availability

The datasets generated during and/or analyzed during this study are not publicly available as they contain participant information but are available from the corresponding author on reasonable request.
