# Supplementary figures and images for: Effectiveness of an Internet-Based Acceptance and Commitment Therapy Intervention for Reducing Psychological Distress in Health Care Professionals: Randomized Controlled Trial
Source: J Med Internet Res. 2024 Dec 18;26:e59093. doi: 10.2196/59093 (PMC11694045; doi:10.2196/59093)

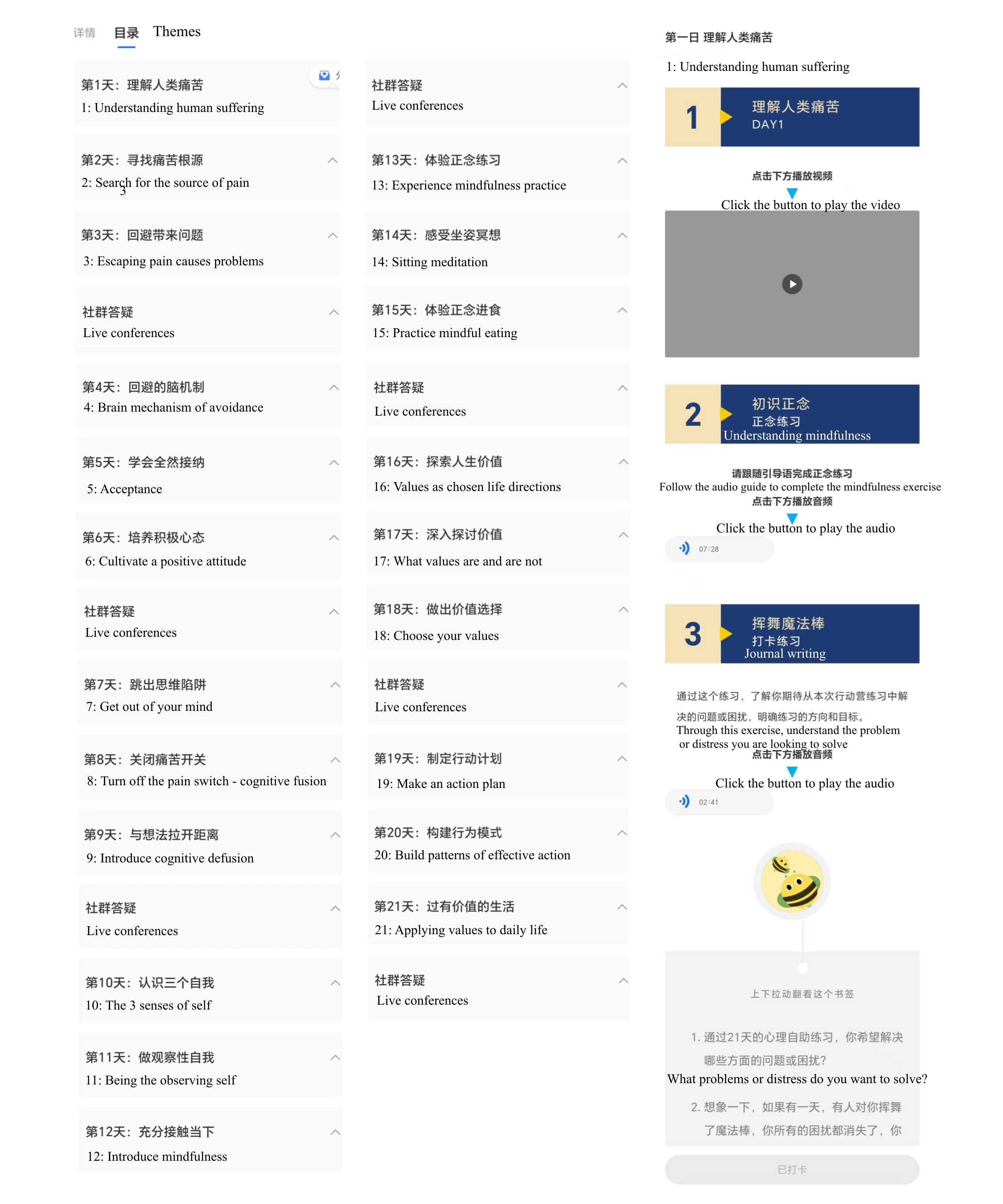

Supplement: Multimedia Appendix 1 [file jmir_v26i1e59093_app1.png]
