# Supplementary material for: Effectiveness of an Internet-Based Acceptance and Commitment Therapy Intervention for Reducing Psychological Distress in Health Care Professionals: Randomized Controlled Trial
Source: J Med Internet Res. 2024 Dec 18;26:e59093. doi: 10.2196/59093 (PMC11694045; doi:10.2196/59093)
Supplement: Multimedia Appendix 2 [file jmir_v26i1e59093_app2.docx]

**Table S1.** Comparison of baseline general information for completed and missed samples.

| Variable | Missing sample (n=40) | Completed sample (n=68) | *t/χ*^2^ | *P* |
| --- | --- | --- | --- | --- |
| **Age，mean(SD)** | 37.28(7.02) | 39.15(7.36) | -1.73 | 0.08 |
| **Gender** |  |  | 3.06 | 0.08 |
| Male | 4(10.0%) | 16(23.5%) |  |  |
| Female | 36(90%) | 52(76.5%) |  |  |
| **Marital status** |  |  | 1.31 | 0.52 |
| Single | 4(10.0%) | 9(13.2%) |  |  |
| Married | 33(82.5%) | 57(83.8%) |  |  |
| Others | 3(7.5%) | 2(2.9%) |  |  |
| **Occupational role** |  |  | 5.63 | 0.06 |
| Physician | 13(32.5%) | 29(42.6%) |  |  |
| Nurse | 21(52.5%) | 37(54.4%) |  |  |
| Other | 6(15.0%) | 2(2.9%) |  |  |
| **Education** |  |  | 0.55 | 0.46 |
| Senior high school | 0(0.0%) | 3(4.4%) |  |  |
| College degree or above | 40(100.0%) | 65(95.6%) |  |  |
| DASS-21，mean(SD) | 20.23(8.60) | 16.31(8.26) | -2.34 | 0.02^a^ |
| Depression，mean(SD) | 6.65(3.89) | 4.91(3.16) | -2.52 | 0.01^a^ |
| Anxiety，mean(SD) | 5.33(3.03) | 4.46(3.13) | -1.84 | 0.07 |
| Stress，mean(SD) | 8.25(3.37) | 6.94(3.17) | -2.30 | 0.02^a^ |
| MBI-GS，mean(SD) | 2.74(0.86) | 2.35(0.68) | -2.58 | 0.01^a^ |
| CompACT，mean(SD) | 33.40(9.56) | 30.13(8.63) | -1.83 | 0.07 |
| Openness to Experience，mean(SD) | 11.90(5.84) | 10.63(5.29) | -1.42 | 0.16 |
| Behavioral Awareness，mean(SD) | 10.45(5.49) | 8.79(4.89) | -0.85 | 0.40 |
| Valued Action，mean(SD) | 11.05(5.17) | 10.71(5.17) | -1.55 | 0.12 |

a The *P* value was statistically significant.
