# Supplementary material for: Effectiveness of an Internet-Based Acceptance and Commitment Therapy Intervention for Reducing Psychological Distress in Health Care Professionals: Randomized Controlled Trial
Source: J Med Internet Res. 2024 Dec 18;26:e59093. doi: 10.2196/59093 (PMC11694045; doi:10.2196/59093)
Supplement: Multimedia Appendix 3 [file jmir_v26i1e59093_app3.pdf]

CONSORT-EHEALTH Checklist V1.6.2 Report

Manuscript Number 59093

(based on CONSORT-EHEALTH V1.6), available at [http://tinyurl.com/consort-ehealth-v1-6].

Date completed

10/4/2024 19:28:35

by

Mingzhong Xu

The effectiveness of an Internet-based acceptance and commitment therapy intervention for reducing psychological distress in healthcare professionals: A randomized controlled trial

TITLE

1a-i) Identify the mode of delivery in the title

Internet-based acceptance and commitment therapy intervention

1a-ii) Non-web-based components or important co-interventions in title

We don't have non-web-based components or important co-interventions

1a-iii) Primary condition or target group in the title

psychological distress in healthcare professionals

ABSTRACT

1b-i) Key features/functionalities/components of the intervention and comparator in the METHODS section of the ABSTRACT

From October 2022 to January 2023, 108 HCPs were recruited online and randomized into a 6-week iACT intervention program with therapist support (n=54) or waitlist control (WLC) group (n=54). The intervention included 21 self-guided sessions combining teaching videos, mindfulness practices, and journal writing, followed by seven live conferences to share experiences and discuss questions, all conducted online.

1b-ii) Level of human involvement in the METHODS section of the ABSTRACT

7 live conferences to share experiences and discuss questions.

1b-iii) Open vs. closed, web-based (self-assessment) vs. face-to-face assessments in the METHODS section of the ABSTRACT

Primary outcomes and secondary outcomes were collected using the Sojump platform

1b-iv) RESULTS section in abstract must contain use data

All attended at least 2 sessions, and 25 attended all 28 sessions. On average, participants attended 20 (71%) sessions.

1b-v) CONCLUSIONS/DISCUSSION in abstract for negative trials

N/A

INTRODUCTION

2a-i) Problem and the type of system/solution

Considering the high prevalence and severe outcomes of psychological distress and burnout among HCPs, it is both urgent and critical to conduct effective interventions to address these problems among HCPs.

2a-ii) Scientific background, rationale: What is known about the (type of) system

However, the application of ACT in HCPs is limited due to the heavy workload and limited free time of HCPs, making it challenging for them to attend face-to-face ACT interventions. These limitations could be addressed by internet-delivered ACT (iACT), which has gained increasing popularity due to its easy accessibility, greater convenience, and cost-effectiveness.

Does your paper address CONSORT subitem 2b?

There is limited evidence of its effectiveness in improving HCPs' mental health in China. Thus, this study aimed to examine the effectiveness of an iACT intervention program in reducing psychological distress (stress, anxiety, and depression) and burnout and improving psychological reflexibility among HCPs in China.

METHODS

3a) CONSORT: Description of trial design (such as parallel, factorial) including allocation ratio

A parallel randomized controlled study with four repeated measures was conducted among HCPs

3b) CONSORT: Important changes to methods after trial commencement (such as eligibility criteria), with reasons

There were no important changes to methods after trial commencement

3b-i) Bug fixes, Downtimes, Content Changes

N/A

4a) CONSORT: Eligibility criteria for participants

The inclusion criteria of participants included (1) age≥18 years; (2) registered HCPs with formal licenses issued by the National Health Department and with at least one year of working experience; (3) able to use the Internet and WeChat. The exclusion criteria were: (1) participants who were off duty due to sick leave, maternal leave, or other reasons during the study period; (2) participants who had a prior diagnosis of severe mental illness; (3)participants with severe suicidal ideation as assessed by the last item score of the Depression Anxiety and Stress Scales – 21(DASS-21) ≥ 3 or suicidal behaviors; (4) participants who had received oral psychotropic drug or psychotherapy treatment within the past three months.

4a-i) Computer / Internet literacy

N/A

4a-ii) Open vs. closed, web-based vs. face-to-face assessments:

Potential participants were recruited online through advertisements on WeChat, and those interested could scan a Quick Response (QR) code to complete a Web-based screening questionnaire to determine their eligibility. WeChat is a multifunctional messaging app that offers messaging, voice calls, video calls, mobile payments, mini-programs, and a wide range of other features for daily use [32].

4a-iii) Information giving during recruitment

Potential participants were recruited through advertisements on WeChat.

4b) CONSORT: Settings and locations where the data were collected

All assessments were conducted through questionnaires on the Sojump website (http://www.sojump.com/). Sojump, also named Wenjuanxing in Chinese, is one of the largest online survey platforms in China [33]. It provides multiple free functions related to surveys, such as questionnaire development and distribution, data analysis, and result reporting, similar to Qualtrics.com or SurveyMonkey.com in the United States [33].

4b-i) Report if outcomes were (self-)assessed through online questionnaires

All assessments were conducted through questionnaires on the Sojump website (http://www.sojump.com/). Sojump, also named Wenjuanxing in Chinese, is one of the largest online survey platforms in China [33]. It provides multiple free functions related to surveys, such as questionnaire development and distribution, data analysis, and result reporting, similar to Qualtrics.com or SurveyMonkey.com in the United States [33].

4b-ii) Report how institutional affiliations are displayed

N/A

5) CONSORT: Describe the interventions for each group with sufficient details to allow replication, including how and when they were actually administered

5-i) Mention names, credential, affiliations of the developers, sponsors, and owners

N/A

5-ii) Describe the history/development process

The intervention was tested among the general population in the early stages, and positive feedback was received.

5-iii) Revisions and updating

N/A

5-iv) Quality assurance methods

N/A

5-v) Ensure replicability by publishing the source code, and/or providing screenshots/screen-capture video, and/or providing flowcharts of the algorithms used

Multimedia Appendix 1

**5-vi) Digital preservation**		
N/A		
**5-vii) Access**		
The six-week iACT intervention was offered through the Little Program on WeChat, and participants need to log in to the Little Program before using it. The flowchart of the specific intervention module and screenshots of the WeChat Mini Program are shown in the supplementary file Fig.1.		
**5-viii) Mode of delivery, features/functionalities/components of the intervention and comparator, and the theoretical framework**		
The iACT program contained 28 sessions. The first 21 self-guided sessions were recorded by a psychologist and a meditation instructor, with each session unlocked every other day. Each session included a 10-15 minute recorded teaching video of a therapist, a 5-8 minute audio recording of guided mindfulness practice, and a journal writing task. The journals can be public or private between the "instructor" and the "student." Participants received a WeChat message on the morning of practice day about the assigned session and practice assignment, which needed to be completed within 48 hours. After watching the video, listening to the audio, and submitting the journal, the participants were considered to have completed one session. After that, seven web-based live conferences were held on days 6, 12, 18, 24, 30, 36, and 42 by an ACT therapist to review lessons, answer questions, and discuss difficult experiences.		
**5-ix) Describe use parameters**		
The iACT program contained 28 sessions.		
**5-x) Clarify the level of human involvement**		
Seven web-based live conferences were held on days 6, 12, 18, 24, 30, 36, and 42 by an ACT therapist.		
**5-xi) Report any prompts/reminders used**		
Participants received a WeChat message on the morning of practice day about the assigned session and practice assignment, which needed to be completed within 48 hours.		
**5-xii) Describe any co-interventions (incl. training/support)**		
All interventionists received standard training in ACT and had rich experience in delivering iACT. They provided the intervention following a highly standardized treatment protocol and were supervised during the intervention.		
**6a) CONSORT: Completely defined pre-specified primary and secondary outcome measures, including how and when they were assessed**		
Primary outcome measure<br>The Depression Anxiety and Stress Scale – 21(DASS-21) [37] was used to assess HCPs' psychological distress with three subscales: depression, anxiety, and stress.<br>Secondary outcome measure<br>Burnout was measured by the Maslach Burnout Inventory-General Survey (MBI-GS) developed by Maslach et al. and adapted by Chinese scholar Li Chaoping [40].<br>Psychological flexibility was assessed using the Comprehensive Assessment of ACT Processes (CompACT) as a process measurement for ACT.		
**6a-i) Online questionnaires: describe if they were validated for online use and apply CHERRIES items to describe how the questionnaires were designed/deployed**		
N/A		
**6a-ii) Describe whether and how "use" (including intensity of use/dosage) was defined/measured/monitored**		
The number of sessions completed by participants is recorded in the background.		
**6a-iii) Describe whether, how, and when qualitative feedback from participants was obtained**		
The journals can be public or private between the "instructor" and the "student."		
**6b) CONSORT: Any changes to trial outcomes after the trial commenced, with reasons**		
All assessments were conducted through questionnaires on the Sojump website (http://www.sojump.com/). Sojump, also named Wenjuanxing in Chinese, is one of the largest online survey platforms in China [33]. It provides multiple free functions related to surveys, such as questionnaire development and distribution, data analysis, and result reporting, similar to Qualtrics.com or SurveyMonkey.com in the United States [33].		
**7a) CONSORT: How sample size was determined**		
**7a-i) Describe whether and how expected attrition was taken into account when calculating the sample size**		
The effect size was set at 0.24, α was set at .05, and the power was set at 0.80, an attrition rate of 22.		
**7b) CONSORT: When applicable, explanation of any interim analyses and stopping guidelines**		
Primary outcome measure<br>The Depression Anxiety and Stress Scale – 21(DASS-21) [37] was used to assess HCPs' psychological distress with three subscales: depression, anxiety, and stress.<br>Secondary outcome measure<br>Burnout was measured by the Maslach Burnout Inventory-General Survey (MBI-GS) developed by Maslach et al. and adapted by Chinese scholar Li Chaoping [40].<br>Psychological flexibility was assessed using the Comprehensive Assessment of ACT Processes (CompACT) as a process measurement for ACT.		
**8a) CONSORT: Method used to generate the random allocation sequence**		
After providing electronic informed consent, the participants were randomly allocated 1:1 to the iACT group or the waitlist control (WLC) group. A simple randomization method was used to randomize the participants. Researchers who were blinded to the study generated random sequences on a website and assigned participants to the appropriate group. In addition, data collectors were unaware of the group assignments throughout the study period.		
**8b) CONSORT: Type of randomisation; details of any restriction (such as blocking and block size)**		
A simple randomization method was used to randomize the participants.		
**9) CONSORT: Mechanism used to implement the random allocation sequence (such as sequentially numbered containers), describing any steps taken to conceal the sequence until interventions were assigned**		
A simple randomization method was used to randomize the participants. Researchers who were blinded to the study generated random sequences on a website and assigned participants to the appropriate group. In addition, data collectors were unaware of the group assignments throughout the study period.		
**10) CONSORT: Who generated the random allocation sequence, who enrolled participants, and who assigned participants to interventions**		
Researchers who were blinded to the study generated random sequences on a website and assigned participants to the appropriate group.		
**11a) CONSORT: Blinding - If done, who was blinded after assignment to interventions (for example, participants, care providers, those assessing outcomes) and how**		
**11a-i) Specify who was blinded, and who wasn't**		
Researchers who were blinded to the study generated random sequences on a website and assigned participants to the appropriate group. In addition, data collectors were unaware of the group assignments throughout the study period.		
**11a-ii) Discuss e.g., whether participants knew which intervention was the "intervention of interest" and which one was the "comparator"**		
All participants provided electronic informed consent before participation.		
**11b) CONSORT: If relevant, description of the similarity of interventions**		
this item is usually not relevant for ehealth trials as it refers to similarity of<br>a placebo or sham intervention to a active medication/intervention		
**12a) CONSORT: Statistical methods used to compare groups for primary and secondary outcomes**		
Generalized estimating equations (GEEs) were used to compare the outcomes (DASS-21, MBI-GS, and CompACT score changes) between the two groups and assess the group, time, and group-by-time interaction effects. Assessments conducted at baseline (T0), week 2(T1), week 6 (T2), and week 10 (T3) were included as outcomes in the GEE analysis, with 0.2, 0.5, and 0.8 representing a small, medium, and large effect, respectively.		
**12a-i) Imputation techniques to deal with attrition / missing values**		
There were no significant differences in baseline characteristics between the intervention group and the control group (Table 2) and between the completed sample and the missing sample (supplementary file S1).		
**12b) CONSORT: Methods for additional analyses, such as subgroup analyses and adjusted analyses**		
A subgroup analysis was performed using the independent samples t-test to explore the gender differences in the intervention effects. In order to test whether participants with more severe psychological distress benefit more from the iACT program, we conducted a sensitive analysis on participants with a DASS-21 score ≥20 (65th percentile) at baseline.		
RESULTS		
**13a) CONSORT: For each group, the numbers of participants who were randomly assigned, received intended treatment, and were analysed for the primary outcome**		

A total of 173 HCPs were recruited, among whom 108 (28 physicians, 35 nurses, and 5 support staff) were included and randomized into the iACT group (n=54) and the WLC group (n=54). Among the 108 participants, 99 completed at least one assessment (91.7%), and 68 (63.0%) completed the follow-up assessment at week 10, including 35 in the iACT group and 33 in the WLC group (Fig.1).

13b) CONSORT: For each group, losses and exclusions after randomisation, together with reasons

A total of 173 HCPs were recruited, among whom 108 (28 physicians, 35 nurses, and 5 support staff) were included and randomized into the iACT group (n=54) and the WLC group (n=54). Among the 108 participants, 99 completed at least one assessment (91.7%), and 68 (63.0%) completed the follow-up assessment at week 10, including 35 in the iACT group and 33 in the WLC group (Fig.1).

13b-i) Attrition diagram

Fig 1

14a) CONSORT: Dates defining the periods of recruitment and follow-up

No critical “secular events” fell into the study period,

14a-i) Indicate if critical “secular events” fell into the study period

N/A

14b) CONSORT: Why the trial ended or was stopped (early)

the trial ended when the study finished

15) CONSORT: A table showing baseline demographic and clinical characteristics for each group

At baseline, the participants had a mean age of 38.45 (SD 7.26) years, and 81.5% (88/108) were female. Their mean scores for depression, anxiety, stress, burnout, and psychological flexibility were 5.56 (SD 3.53),4.78 (SD 3.10),7.43 (SD 3.29),2.49 (SD 0.77), and 31.34 (SD 9.08), respectively (Table 1).

15-i) Report demographics associated with digital divide issues

At baseline, the participants had a mean age of 38.45 (SD 7.26) years, and 81.5% (88/108) were female. Their mean scores for depression, anxiety, stress, burnout, and psychological flexibility were 5.56 (SD 3.53),4.78 (SD 3.10),7.43 (SD 3.29),2.49 (SD 0.77), and 31.34 (SD 9.08), respectively (Table 1).

16a) CONSORT: For each group, number of participants (denominator) included in each analysis and whether the analysis was by original assigned groups

16-i) Report multiple “denominators” and provide definitions

Among the 108 participants, 99 completed at least one assessment (91.7%), and 68 (63.0%) completed the follow-up assessment at week 10, including 35 in the iACT group and 33 in the WLC group (Fig.1).

16-ii) Primary analysis should be intent-to-treat

Fig. 1

17a) CONSORT: For each primary and secondary outcome, results for each group, and the estimated effect size and its precision (such as 95% confidence interval)

Primary outcomes
As shown in Table 3, the GEE model revealed a statistically significant group effect and group-by-time interaction effect on the DASS-21 score.

Secondary Outcomes
Table 3 also showed statistically significant group effect, time effect, and group-by-time interaction effects on burnout and psychological flexibility.

17a-i) Presentation of process outcomes such as metrics of use and intensity of use

Of the 54 participants in the iACT group, all attended at least 2 sessions.

17b) CONSORT: For binary outcomes, presentation of both absolute and relative effect sizes is recommended

The intergroup effect sizes for psychological distress at week 6 and week 10 were 0.82 (95% CI 0.39-1.26) and 1.08 (95% CI 0.57-1.59), respectively.
The intergroup effect sizes for burnout at week 6 and week 10 were 1.42 (95% CI 0.95-1.89) and 1.52 (95% CI 0.98-2.06), respectively.
The intergroup effect sizes for psychological flexibility at week 6 and week 10 were 1.23 (95% CI 0.77-1.69) and 1.15 (95% CI 0.64-1.66), respectively.

18) CONSORT: Results of any other analyses performed, including subgroup analyses and adjusted analyses, distinguishing pre-specified from exploratory

Sub-group analysis
Considering the large proportion of females in the final sample, we further conducted a sex-based subgroup analysis to test if there were any significant gender differences in the intervention effects. Table 4 shows the results of the independent samples t-tests to assess the primary and secondary outcomes. There were no statistical gender differences in all outcomes, including the DASS-21, MBI-GS, and CompACT scores.

Sensitivity analysis
To examine the effect of the interventions on participants who exhibited higher levels of distress, we conducted a sensitivity analysis on the participants with a DASS-21 score ≥20 (65th percentile) at baseline (21 in the iACT group and 20 in the WLC group). As shown in Table 4, the scores of the DASS-21 total scale and its three subscales increased in the iACT group but decreased in the WLC group. The iACT group showed more substantial changes in DASS-21 score at weeks 2, 6, and 10 (β= -7.91, -8.44, -11.11, respectively) than the WLC group. At follow-up, the ACT group had more participants who no longer met the DASS-21 criterion for significant distress than the WLC group (77 % v.s. 64 %). Regarding burnout, compared to the WLC group, the iACT group showed more remarkable changes in burnout scores from T0 to T3 (β= -0.74), from T0 to T2 (β= -0.74), and from T0 to T1 (β= -0.48). As for psychological flexibility, the ACT group showed more substantial changes in CompACT score at weeks 2, 6, and 10 (β= -6.41, -9.35, -8.30, respectively), also reflected in its three dimensions of openness to experience, behavioral awareness, and valued action.

18-i) Subgroup analysis of comparing only users

Of the 54 participants in the iACT group, all attended at least 2 sessions.

19) CONSORT: All important harms or unintended effects in each group

There was no important harms or unintended effects in each group

19-i) Include privacy breaches, technical problems

N/A

19-ii) Include qualitative feedback from participants or observations from staff/researchers

N/A

DISCUSSION

20) CONSORT: Trial limitations, addressing sources of potential bias, imprecision, multiplicity of analyses

20-i) Typical limitations in ehealth trials

First, the sample was predominantly female, which may limit the generalization of the findings to males. However, subsequent sex-based subgroup analysis showed no significant differences. Future studies may consider recruiting a more gender-balanced sample to validate our results. Second, potential sampling bias may exist due to the self-selective nature of the online recruitment strategy. HCPs with higher levels of psychological distress who may benefit more from the intervention may be more likely to actively participate in the study, leading to more significant observed intervention effects. While large effect sizes were observed for decreases in general distress in participants and in those who exhibited higher levels of distress, only tentative conclusions can be made given the potential sampling bias.

21) CONSORT: Generalisability (external validity, applicability) of the trial findings

21-i) Generalizability to other populations

To address the research-to-practice gap and improve the adoption of the iACT program, we need to overcome those barriers using a multilevel approach.

21-ii) Discuss if there were elements in the RCT that would be different in a routine application setting

A long intervention duration with more treatment sessions to ensure a large effect size.

22) CONSORT: Interpretation consistent with results, balancing benefits and harms, and considering other relevant evidence

22-i) Restate study questions and summarize the answers suggested by the data, starting with primary outcomes and process outcomes (use)

Our results demonstrated the effectiveness of the iACT intervention in reducing HCPs' burnout and psychological distress, including stress, anxiety, and depression, as well as improving their psychological flexibility. The effect was more substantial among HCPs with higher baseline psychological distress. Following six weeks of iACT intervention, the iACT group had more significant decreases in psychological distress and burnout and a more significant increase in psychological flexibility than the WLC group. The effects persisted at follow-up assessments one month later. These results are consistent with a large amount of evidence demonstrating the efficacy of iACT in individuals with different psychological conditions [44-47]. A meta-analysis found that iACT demonstrated small yet significant and enduring effects on depression and anxiety compared to control groups[48].

22-ii) Highlight unanswered new questions, suggest future research

More RCT studies with more extended follow-up periods are warranted to evaluate the long-term effectiveness of iACT in the future.

Other information		
**23) CONSORT: Registration number and name of trial registry**		
Trial Registration: Chinese Clinical Trial Register ChiCTR 2400093584		
**24) CONSORT: Where the full trial protocol can be accessed, if available**		
Trial Registration: Chinese Clinical Trial Register ChiCTR 2400093584		
**25) CONSORT: Sources of funding and other support (such as supply of drugs), role of funders**		
This research was supported by the Science Foundation of Changsha City (kq2202039) and the Changsha City Health Medicine Research Project (KJ-B2023100).		
**X26-i) Comment on ethics committee approval**		
N/A		
**x26-ii) Outline informed consent procedures**		
Participants were fully informed of the study ' s objective, procedures, benefits, and potential risks.		
**X26-iii) Safety and security procedures**		
Participation in the study was totally voluntary, and participants could withdraw from the study at any time.		
**X27-i) State the relation of the study team towards the system being evaluated**		

N/A
